# Integration of (*S*)-2,3-oxidosqualene enables *E. coli* to become Iron Man *E. coli* with improved overall tolerance

**DOI:** 10.1186/s13068-023-02444-7

**Published:** 2023-12-10

**Authors:** Wenjie Sun, Yun Chen, Mengkun Li, Syed Bilal Shah, Tianfu Wang, Jin Hou, Linquan Bai, Yan Feng, Zaigao Tan

**Affiliations:** 1 State Key Laboratory of Microbial Metabolism, Shanghai Jiao Tong University, 224 Wenxuan Building800 Dongchuan Road, Minhang District, Shanghai, 200240 China; 2 School of Life Sciences and Biotechnology, Shanghai Jiao Tong University, Shanghai, 200240 China; 3 Department of Bioengineering, Shanghai Jiao Tong University, Shanghai, 200240 China; 4 School of Environmental Science and Engineering, Shanghai Jiao Tong University, Shanghai, 200240 China; 5 State Key Laboratory of Microbial Technology, Shandong University, Qingdao, 266237 Shandong Province China

**Keywords:** Sterols biosynthesis, (*S*)-2,3-oxidosqualene, *E. coli*, Severe stresses, Chemicals production

## Abstract

**Background:**

While representing a model bacterium and one of the most used chassis in biomanufacturing, performance of *Escherichia coli* is often limited by severe stresses. A super-robust *E. coli* chassis that could efficiently tolerant multiple severe stresses is thus highly desirable. Sterols represent a featured composition that distinguishes eukaryotes from bacteria and all archaea, and play a critical role in maintaining the membrane integrity of eukaryotes. All sterols found in nature are directly synthesized from (*S*)-2,3-oxidosqualene. However, in *E. coli*, (*S*)-2,3-oxidosqualene is not present.

**Results:**

In this study, we sought to introduce (*S*)-2,3-oxidosqualene into *E. coli*. By mining and recruiting heterologous enzymes and activation of endogenous pathway, the ability of *E. coli* to synthesize (*S*)-2,3-oxidosqualene was demonstrated. Further analysis revealed that this non-native chemical confers *E. coli* with a robust and stable cell membrane, consistent with a figurative analogy of wearing an “Iron Man’s armor”-like suit. The obtained Iron Man *E. coli* (IME) exhibited improved tolerance to multiple severe stresses, including high temperature, low pH, high salt, high sugar and reactive oxygen species (ROS). In particular, the IME strain shifted its optimal growth temperature from 37 °C to 42–45 °C, which represents the most heat-resistant *E. coli* to the best of our knowledge. Intriguingly, this non-native chemical also improved *E. coli* tolerance to a variety of toxic feedstocks, inhibitory products, as well as elevated synthetic capacities of inhibitory chemicals (e.g., 3-hydroxypropionate and fatty acids) due to improved products tolerance. More importantly, the IME strain was effectively inhibited by the most commonly used antibiotics and showed no undesirable drug resistance.

**Conclusions:**

Introduction of the non-native (*S*)-2,3-oxidosqualene membrane lipid enabled *E. coli* to improve tolerance to various stresses. This study demonstrated the effectiveness of introducing eukaryotes-featured compound into bacteria for enhancing overall tolerance and chemical production.

**Supplementary Information:**

The online version contains supplementary material available at 10.1186/s13068-023-02444-7.

## Introduction

Developing microbial cell factories for the efficient synthesis of chemicals and fuels provides a green, safe and renewable alternative route to traditional petroleum-based techniques. Various microbes have been genetically manufactured for synthesis of a variety of bulk chemicals, fine chemicals, nature products, biofuels and polymers [1]. Among these microbes, *Escherichia coli* has become one of the most used chassis cells in biomanufacturing field owing to its specific advantages, including fast growth, genetic tractability, easy culture, well characterized metabolic background and availability of genetic engineering tools [2]. As such, *E. coli* has been successfully engineered for production of a series of industrially relevant chemicals, including 1,3-propanediol [3],1,4 butandiol [4], isobutanol [5], L-alanine [6], succinate [7] and so on.

However, performances of *E. coli* strains are often limited by severe stresses [8–12]. For instance, cultivation of *E. coli* with high growth rate frequently generates a large amount of biological heat, which makes the growth of *E. coli* ceased at a high temperature (e.g., 45 °C) [13]. Using *E. coli* to produce organic acids often causes the products accumulation with reducing the pH, and *E. coli* cells were rapidly killed under a moderate acidic condition (e.g., pH 4.2) [11]. For maintaining the optimal pH for continued product formation, a base often has to be added, which causes the accumulation of cations (e.g., Na^+^ or K^+^) [14]. While even though low concentration of cations (e.g., 400 mM Na^+^) decreased the specific growth rate of *E. coli* by more than 45% [14]. Cultivation with a high initial sugar concentration is highly desired in batch fermentation for minimizing sugar feeding. However, high sugar concentration often leads to high osmotic pressure, which impairs both the growth and product synthesis of *E. coli*. When cultivated in a high glucose medium (120 g/L), the cell mass and succinate titer of *E. coli* Suc-T110 strain decreased by 37% and 30%, respectively [15]. In addition, *E. coli* often encounters reactive oxygen species (ROS) sourced from either endogenous metabolisms or environments, thus causing oxidative stress and severely inhibiting growth [16].

Therefore, developing robust *E. coli* strains to resist these severe stresses becomes indispensable. However, current engineering strategies heavily rely on exploitation of the potential of *E. coli* alone [17–19]. Not surprisingly, few successes have been achieved from such engineering strategies, as it is unreasonable to deal with different stress factors by exploiting *E. coli* alone. Furthermore, these engineering strategies frequently take effect to only one certain stress rather than multiple stresses encountered by *E. coli* during the real production process [20–22].

Fortunately, over the course of evolution, organisms with distinct advantageous structures have survived in environments [23, 24], which provide us gold mine for mining robust cellular structures and applying them to *E. coli* for constructing a super-robust *E. coli* chassis, which is expected to outcompete the strategy that solely patches its structures. Among the four basic compositions of cells including carbohydrates, nucleic acids, proteins and lipids, lipids are often the easily ignored molecules of the microbial sciences [25]. Actually, lipids have great potentials for serving as robust cellular structures as it is lipids (e.g., phospholipids, glycolipid and cholesterol) that consist of the fundamental structure of cells [26].

**Fig. 1:**
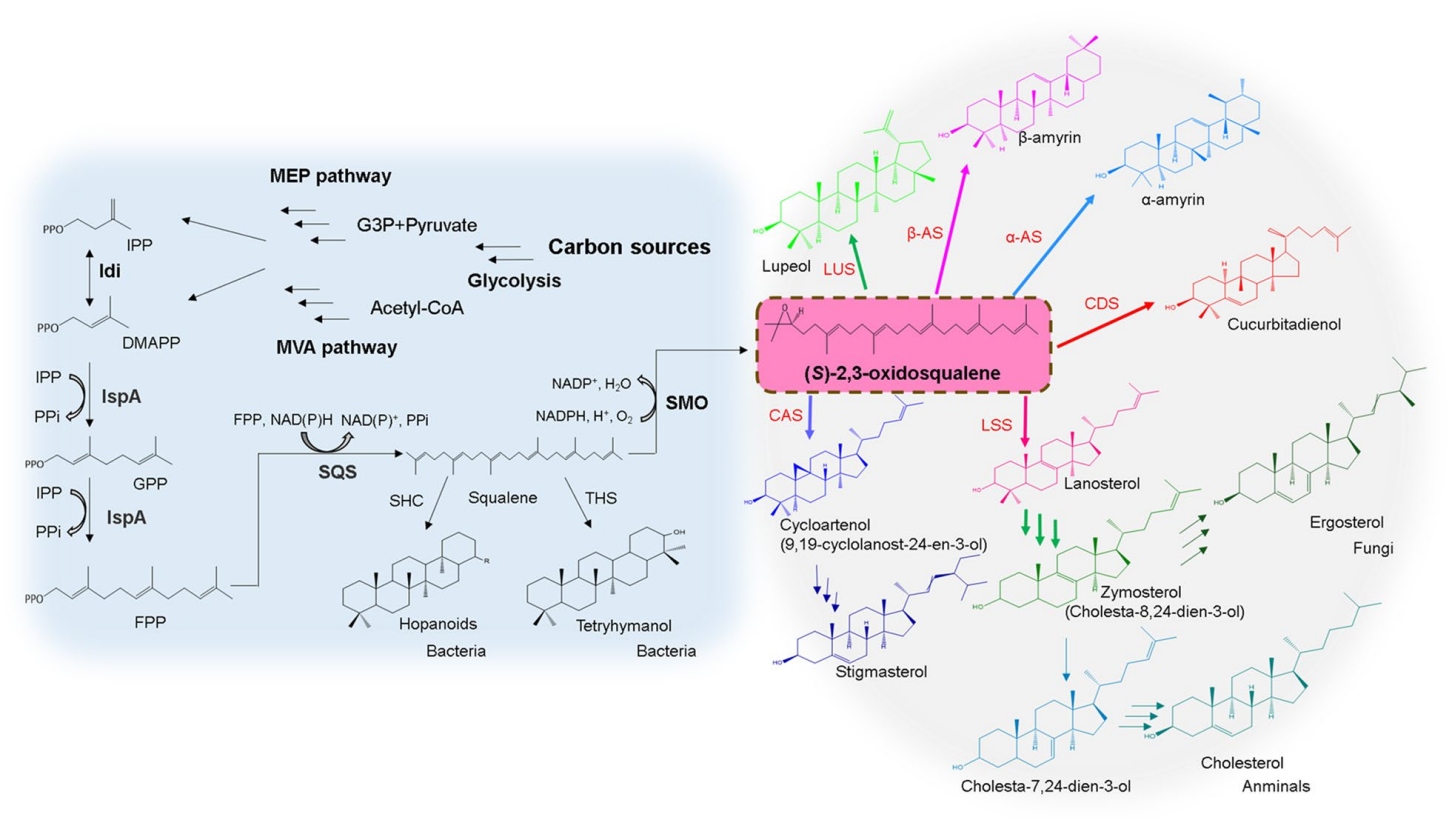
(*S*)-2,3-oxidosqualene serves as the common precursor of synthesis of all types of sterols. Multiple arrows represent more than one reaction step; squalene hopene cyclase: SHC; tetrahymanol synthase: THS. lanosterol synthases: LSS; cyloartenol synthases: CAS; cucurbitadienol synthases: CDS; α-amyrin synthases: α-AS; β-amyrin synthases: β-AS; lupeol synthases: LUS

Sterols are the third lipid class, and also represent a featured lipids that distinguishes eukaryotes from bacteria and archaea [27, 28]. Sterols play a critical role in maintaining the membrane integrity of eukaryotes, and have been deemed as membrane reinforcers [29]. In particular, cholesterol is the major sterol of animals, ergosterol plays a key role in yeast and fungi, and plants usually possess more complex sterol compositions such as stigmasterol and sitosterol [29] (Fig. 1). Sterols found in nature are all directly synthesized from (*S*)-2,3-oxidosqualene [28] (Fig. 1). However, in *E. coli*, the key chemical (*S*)-2,3-oxidosqualene is not present. In this study, we sought to introduce this non-native chemical into the *E. coli*. We show that this lipid conferred *E. coli* with a robust and stable cell membrane. Given the role of the plasma membrane in defining cellular border, maintaining cellular shape, and protecting cellular integrity [30], this remodeling strategy can be compared to wearing an “Iron Man’s armor-like” suit for *E. coli* chassis. Consistent with this analogy, the engineered strain showed an effective increase in the tolerance to multiple adverse stresses, and improved tolerance to a variety of toxic feedstocks, inhibitory products, as well as elevated synthetic capacities of numerous valuable chemicals.

## Results

### Introduction and optimization of (*S*)-2,3-oxidosqualene biosynthesis pathway in *E. coli*

We first sought to design the (*S*)-2,3-oxidosqualene biosynthetic pathway in *E. coli*. In this design, the native methylerythritol phosphate (MEP) pathway converts carbon sources into dimethylallyl pyrophosphate (DMAPP) and isopentenyl pyrophosphate (IPP). Then, farnesyl diphosphate synthase (IspA) catalyzes the condensation reaction between DMAPP and IPP to form farnesyl pyrophosphate (FPP, C15). Two FPPs will be subsequently condensed by squalene synthase (SQS) to form squalene (C30), and then catalyzed by squalene monooxygenase (SMO) to yield (*S*)-2,3-oxidosqualene. However, in *E. coli*, key enzymes SQS and SMO are absent.

We then mined SQS and SMO enzymes, and candidates from the representative eukaryote *Saccharomyces cerevisiae* were first considered. In *S. cerevisiae*, the genes *erg9* and *erg1* encode SQS and SMO enzymes, respectively. ERG9 has been reported to anchor its C-terminal domain to plasma membrane and exposes its catalytic domain to the cytoplasm [31]. To further improve its solubility in *E. coli*, we decided to remove the membrane-anchored domain of ERG9, and this truncation enabled the truncated ERG9 (tERG9) exhibits a relatively high solubility in *E. coli* BL21 (DE3) (Additional file 1: Figure S1).

For SMO, although many canonical protein solubility optimization efforts have been made here, including lowering induction temperature and varying IPTG inducer concentrations [32], the solubility of ERG1 in *E. coli* still remains a challenge. We thus have to turn to mine other SMO candidates. Of numerous enzymes identified from Metacyc and Brenda databases, we selected the SMO from *Methylococcus capsulatus* as it was the first reported prokaryotic squalene monooxygenase [33], sharing a 22% primary sequence identity with ERG1 (Additional file 1: Figure S2). Distinct from ERG1, here we observed that SMO_Mc_ displayed a better solubility in *E. coli* (Additional file 1: Figure S1), which might be owing to the similar intracellular environment shared by *E. coli* and *M. capsulatus* for protein folding.

**Fig. 2:**
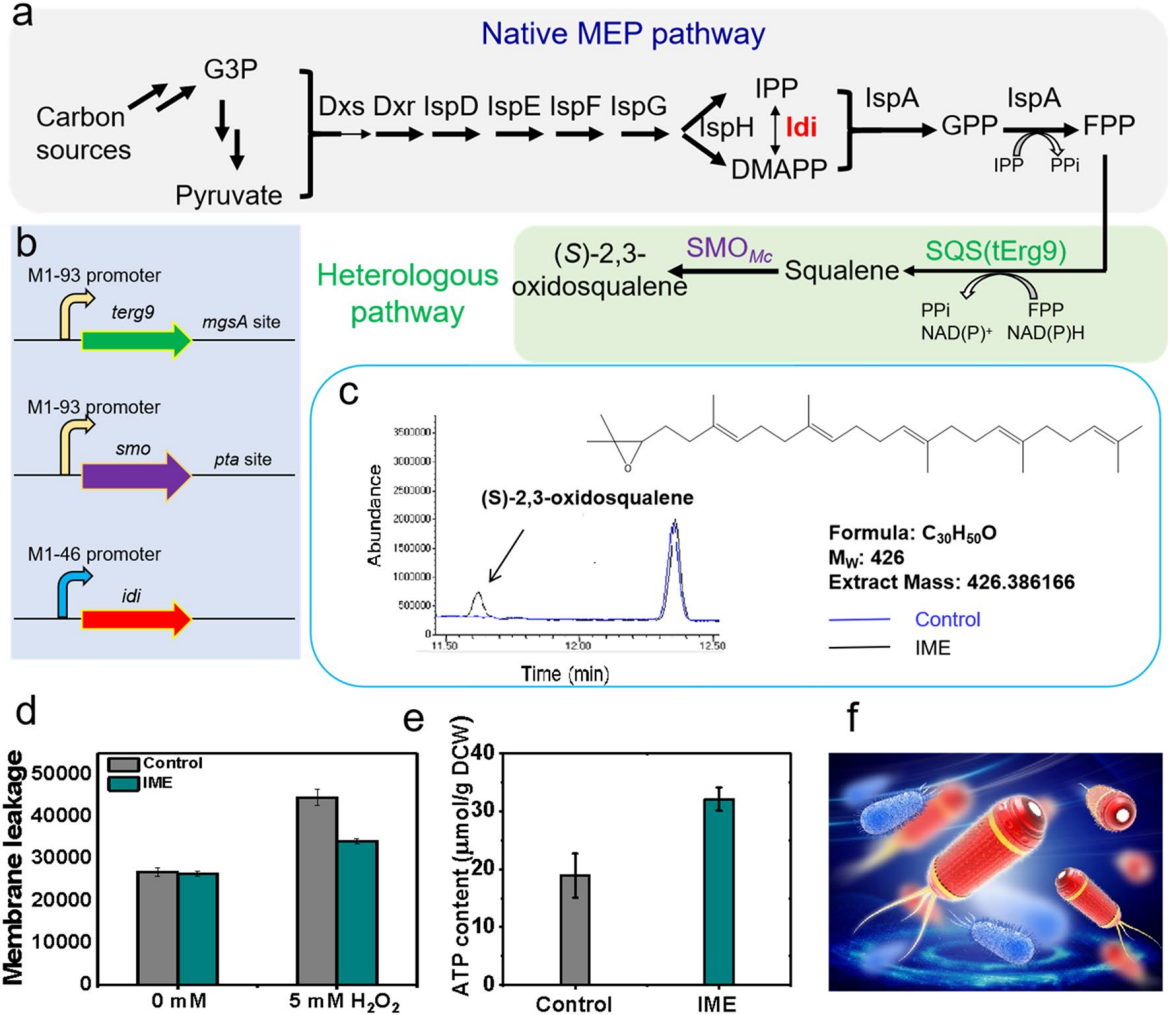
Introduction and optimization of (*S*)-2,3-oxidosqualene biosynthesis pathway in *E. coli* and exhibits decreased membrane leakage and increased intracellular ATP level. **a** Full biosynthetic pathway of (*S*)-2,3-oxidosqualene consisting of native MEP pathway and heterologous pathway; **b** integration of heterologous *S. cerevisiae terg9* gene, *Methylococcus capsulatus smo* gene with M1–93 artificial promoter into genomic DNA of *E. coli* MG1655 at the *mgsA* site and *pta* site, respectively, based on the two pathways genes, the native promoter of *idi* was replaced with artificial promoter M1–46. **c** GC–MS detection of (*S*)-2,3-oxidosqualene for the engineered strains. The engineered strain with the heterologous pathway and promoter replacement of rate-limiting enzyme *idi* synthesized the representative (*S*)-2,3-oxidosqualene, TMS derivative while control strain not. **d** Engineered strain had a 24% decrease in membrane leakage relative to the control strain when challenged with 5 mM H_2_O_2_. Membrane leakage was assessed using the SYTOX Green nucleic acid stain. **e** Engineered strain had a 70% increase in ATP content relative to the control strain during challenge with 5 mM H_2_O_2_. ATP content was assessed using an ATP assay kit (Beyotime). Error bars indicate standard deviation of at least three biological replicates. **f** We termed the engineered strain Iron Man *E. coli* (IME) with a figurative analogy of wearing “Iron Man’s armor”-like suit for *E. coli* cell

After mining enzyme candidates for (*S*)-2,3-oxidosqualene synthesis, we next sought to introduce the two key genes into *E. coli* K-12 MG1655 (Fig. 2a). As plasmid overexpression often causes obvious burdens to host cells, as well as additional and expensive inducers (e.g., IPTG, cumate) have to be added for turning on gene’s expression [34], we thus sought to integrate these required genes into the chromosome DNA of *E. coli*, to express these genes in a plasmid- and inducer-free manner. To do this, a previously developed constitutive M1–93 promoter [35] was placed in front of *terg9,* and the constructed M1–93-*terg9* expression cassette was inserted into the genome of MG1655 at *mgsA* site, followed by insertion of M1–93-*smo*_*Mc*_ expression cassette at *pta* site (Fig. 2b).

However, the presence of (*S*)-2,3-oxidosqualene and other sterols were not detected from the extracted membrane fraction of the engineered SOS1 strain (MG1655 *mgsA*::M1–93-*terg9*, *pta*::M1–93-*smo*_*Mc*_). The MEP pathway converts various carbon sources into DMAPP and IPP precursors, while this pathway is tightly regulated in *E. coli* [36]. We speculated that further activation of MEP pathway will contribute to the (*S*)-2,3-oxidosqualene biosynthesis. To do this, we up-regulated the expression level of the key isopentenyl diphosphate isomerase (Idi) as it has been reported to be the rate-limiting step of MEP pathway. In particular, the native promoter of *idi* was replaced with a strong-strength constitutive promoter M1–46 [36]. Finally, we obtained the engineered *E. coli* strain (MG1655 *mgsA*::M1–93-*terg9*, *pta*::M1–93-*smo*_*Mc*_, M1–46-*idi*). Analogous manipulations were performed to yield the control strain (MG1655 Δ*mgsA*, Δ*pta*, M1–46-*idi*) (Fig. 2b). Presence of (*S*)-2,3-oxidosqualene was successfully detected from the extracted membrane fraction of the engineered strain but not the control strain through gas chromatography analysis (Fig. 2c). The identity of formed (*S*)-2,3-oxidosqualene was further confirmed using the mass spectrometry (*m/z* = 426) (Additional file 1: Figure S3). Further quantitative analysis revealed the content of the (*S*)-2,3-oxidosqualene in the engineered *E. coli* strain was ~ 149 μg/g dry cell weight (DCW). Presence of other sterols, e.g., lanosterol, cholesterol and ergosterol, was not detected from the engineered strain.

### Non-native (*S*)-2,3-oxidosqualene greatly alters membrane properties of *E. coli*

As (*S*)-2,3-oxidosqualene can intercalate into phospholipid bilayers and modulate cell membrane properties of eukaryotic cells, we wondered whether (*S*)-2,3-oxidosqualene can also alter the cell membrane properties of the engineered strain. Prior studies revealed that membrane leakage was a primary damage issue when microorganisms were challenged with stresses [37–39]. We thus first evaluated the membrane leakage in the control and engineered strains under stresses. To do this, a representative membrane-damaging chemical H_2_O_2_ [40] was employed and added. Besides, the SYTOX Green nucleic acid stain [37] was also added to identify cells with leaked cell membrane [37].

Our results showed that in the absence of H_2_O_2_, the control and engineered strains showed no difference in membrane leakage, with SYTOX permeability values being approximately 2.60 × 10^5^ (Fig. 2d). During challenge with 5 mM H_2_O_2_, the SYTOX permeability value for the control strain greatly increased by 70% to 4.45 ± 0.19 × 10^5^. However, under the same condition, the SYTOX permeability value for the engineered strain only slightly increased by 30% to 3.42 ± 0.06 × 10^5^. There results demonstrated that, (*S*)-2,3-oxidosqualene did lead to a significant decrease in cell membrane leakage of engineered *E. coli* strain when challenged with stresses.

Besides cell membrane, we also investigated the intracellular ATP content after introducing (*S*)-2,3-oxidosqualene into *E. coli*, as ATP is the versatile energy compound that provides power to drive many fitness maintaining processes in living cells [41]. During challenge with 5 mM H_2_O_2_, we observed that the average ATP content of the engineered strain was 32.1 ± 2.0 μmol/mg DCW, which was 70% higher than that (*P* = 0.001) of the control (18.9 ± 3.8 μmol/mg DCW) (Fig. 2e). In summary, we believe that both the decrease in cell membrane leakage and the increase in intracellular ATP content could contribute to the improved robustness of engineered strain, consistent with a figurative analogy of wearing an “Iron Man’s armor”-like suit for *E. coli* strain. We thus termed the engineered strain Iron Man *E. coli* (IME) (Fig. 2f).

### The IME strain significantly increased tolerance to adverse environmental stresses

**Fig. 3:**
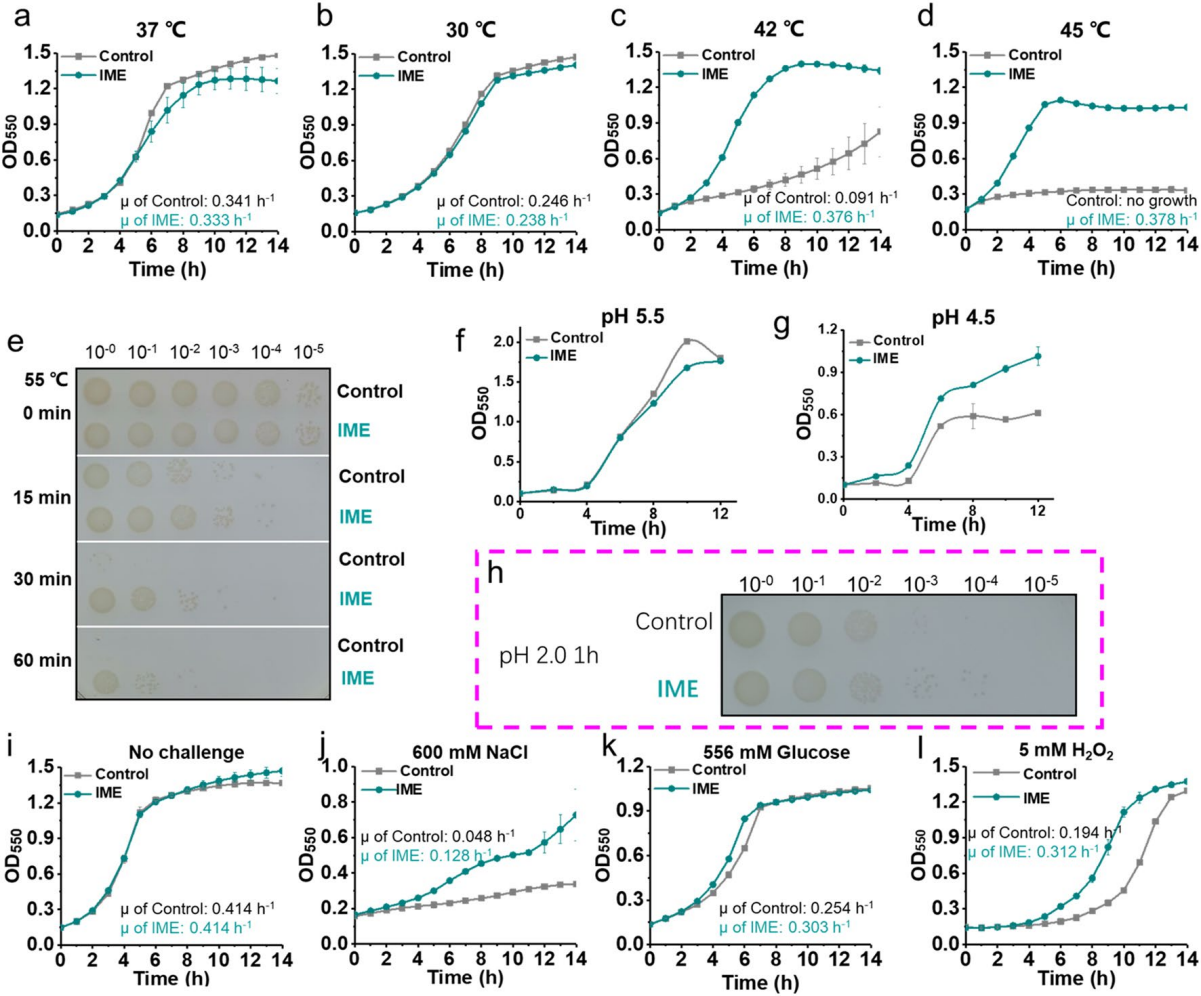
Synthesis of (*S*)-2,3-oxidosqualene in *E. coli* strain and resistance to adverse environmental stresses. **a–d** IME strain had improved thermotolerance, shifted its optimal growth temperature from 37 °C to 42–45 °C. **e** IME strain had improved tolerance to heat shock. **f–h** IME strain showed acid-resistant phenotype. **i**, no challenge. **j–l** IME strain showed improved tolerance to other adverse environmental stresses such as high salt condition, high sugar condition and ROS (H_2_O_2_). Error bars indicate standard deviation of at least three biological replicates

We next assessed phenotypes of the IME strain with (*S*)-2,3-oxidosqualene. Under cultivation at 37 °C in MOPS + 2% (wt/v) glucose minimal medium, the IME strain showed a slight growth decline compared with the control strain, with its specific growth rate (μ, 0.333 h^−1^) being comparable to the control strain (0.341 h^−1^, *P* > 0.05) (Fig. 3a). The similar trend was also observed when the strains were cultivated at a lower temperature 30 °C (Fig. 3b). We speculated that this slight growth decline might be due to the higher protein burden for overexpression of Idi of MEP pathway and heterologous SMO and SQS enzymes (Additional file 1: Figure S4). Intriguingly, upon cultivation at 42 °C, IME strain showed a threefold increase in its μ (0.376 h^−1^) as compared to the control strain (0.091 h^−1^, *P* = 0.01) (Fig. 3c). Under cultivation at a much higher temperature of 45 °C, the control strain cannot grow up, which is consistent with the lethal temperature levels of *E. coli* reported in previous studies [42] (Fig. 3d). Intriguingly, the IME strain grew rapidly under 45 °C, with its μ reaching 0.378 h^−1^ (Fig. 3d). It seems that the introduction of (*S*)-2,3-oxidosqualene enables the IME strain to shift its optimal growth temperature from 37 °C to 42–45 °C. We further assessed the survival capacities of *E. coli* strains after heat shock under an extreme high temperature condition (e.g., 55 °C). In particular, the control strain cannot survive under 55 °C treatment for as short as 30 min, while the IME strain can still survive under 55 °C treatment even for 60 min (Fig. 3e). To the best of our knowledge, this represents the most heat-resistant *E. coli* strain [13, 43, 44].

We next assessed acid-resistant phenotype of the IME strain. Under cultivation pH at 5.5 in MOPS + 2% (wt/v) glucose minimal medium, IME strain showed a slight growth decline compared with the control strain (Fig. 3f). We deemed this might result from the fact that pH at 5.5 merely represents a moderate acidic condition and will not greatly impair the fitness of *E. coli*. Therefore, we further decreased the cultivation pH to 4.5, to create an extreme acidic condition. Intriguingly, the engineered strain showed a better growth under this condition, with its cell mass (OD _550_ ~ 1.0) improving by 67% as compared to the control strain (OD _550_ ~ 0.6, P = 0.01) (Fig. 3g). We further assessed the survival capacities of *E. coli* strains after extreme acidic shock. In particular, under treatment at pH 2.0 for 1 h, the survival capacity of IME strain displayed at least tenfold higher than that of control strain (Fig. 3h), which further confirmed the increased acid-resistant phenotype of the IME strain.

We further evaluated the tolerance of IME strain to other adverse cultivation stresses, including high salt, high sugar and reactive oxygen species (ROS) (Fig. 3j-–l). Specifically, under a high salt condition with 600 mM NaCl (~ 34.8 g/L), growth of the control strain was greatly inhibited and its biomass only reached OD_550_ ~ 0.36 in 16 h, while OD_550_ of IME strain reached by 2.3-fold to 0.82 in the same time (Fig. 3j). Moreover, under a high sugar condition with 556 mM glucose (~ 100 g/L), the μ of IME strain reached up to 0.303 h^−1^, which increases by 19% than that of the control strain (0.254 h^−1^, *P* < 0.05) (Fig. 3k). For ROS, in the presence of 5 mM H_2_O_2_, the μ of the IME strain (0.312 h^−1^) increased by 60% over the control strain (0.194 h^−1^) (*P* = 0.03) (Fig. 3l). These results demonstrated that the IME strain did improve tolerance to multiple adverse environmental conditions.

### The IME strain improves tolerance to inhibitors in lignocellulose-derived feedstocks

**Fig. 4:**
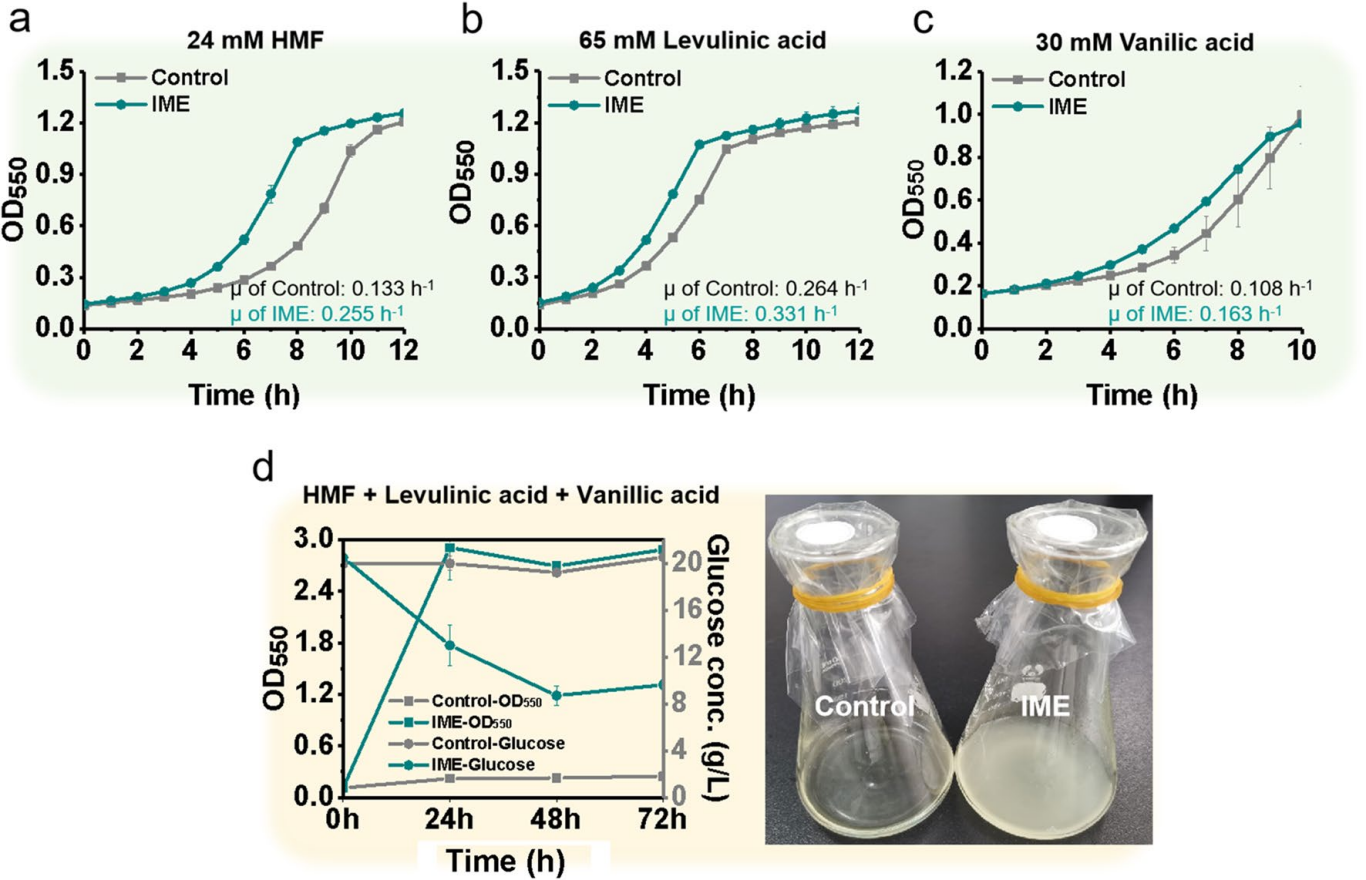
IME improved tolerance to inhibitors in lignocellulose-derived feedstocks. **a–c** IME had improved tolerance to inhibitors existing in the hydrolysate of lignocellulose (HMF, levulinic acid and vanillic acid). Growth curves were recorded in MOPS + 2% glucose medium with different inhibitors in a clear bottom 96-well plate at 37 °C, pH 7.0. **d** IME showed increased cell mass and glucose consumption with addition of representative inhibitors existing in the hydrolysate of lignocellulose. Both control and IME strains were cultivated in MOPS + 2% (w/v) glucose mineral salt medium containing 16 mM HMF (2 g/L), 43 mM levulinic acid (5 g/L), and 6 mM vanillic acid (0.5 g/L) in shake flasks at 37 °C and an initial pH of 7.0. Error bars indicate standard deviation of at least three biological replicates. HMF, hydroxymethylfurfural

Lignocellulose-derived feedstocks are a class of important renewable resource for biomanufacturing [45]. However, inhibitors existing in hydrolysate of lignocellulose impair fitness of microbes [46]. There are three main classes of inhibitors in dilute acid-treated lignocellulose feedstock: furans, weak carboxylic acids and phenolic monomers [47]. For cost-effective utilization of lignocellulosic biomass, construction of these inhibitors-resistant strains is needed. Here, we observed that IME strain improved tolerance to most of these inhibitors (Fig. 4). Most dramatically, IME strain had a 92% increase in its specific growth rate (0.255 h^−1^) relative to the control strain (0.133 h^−1^) in the presence of 24 mM hydroxymethylfurfural (HMF) (~ 3 g/L) (Fig. 4a). For weak carboxylic acids, IME strain displayed a 25% higher specific growth rate (0.331 h^−1^) relative to the control strain (0.264 h^−1^) during challenge with 24 mM levulinic acid (7.5 g/L) (Fig. 4b). For phenolic monomers, IME strain showed a 50.4% increase in specific growth rate (0.108 h^−1^) relative to the control strain (0.163 h^−1^) when challenged with 24 mM vanillic acid (2.5 g/L) (Fig. 4c).

Considering that IME improved tolerance to toxic feedstocks, we thus wondered whether it can be applied for utilization of toxic feedstocks. To this end, we selected utilization of lignocellulose hydrolysate as a demonstration. Specifically, we simulated the existence of main classes of inhibitors and added three representative inhibitors, namely, HMF, levulinic acid, and vanillic acid to MOPS + 2% (wt/v) glucose minimal salts medium at final concentrations of 16 mM (2 g/L), 43 mM (5 g/L) and 6 mM (0.5 g/L), respectively. When cultured in this mimic of lignocellulose hydrolysate, the control strain cannot grow up any more, with zero glucose utilization. In contrast, IME strain showed a rapid growth, with biomass reaching OD_550_ at ~ 3.0 and consuming up to ~ 10 g/L of glucose in only 24 h (Fig. 4d). To the best of our knowledge, this represents the fastest growth rate and highest glucose consumption among all *E. coli* strains under representative inhibitors in hydrolysate of lignocellulose in minimal medium [48–51].

### The IME strain improves tolerance and production of inhibitory products

Among a variety of interesting products, organic acids have received increasing attentions due to not only their growing utilization in food, lubricant, preservative and fuel industries, but also great potential as platform chemicals for synthesis of various biodegradable polymers [52]. However, as with other biorenewable chemicals, high amount of organic acids compromises both the growth and performance of microbial factories [53]. Here, we continued to evaluate performance of IME strain to resisting organic acids.

**Fig. 5:**
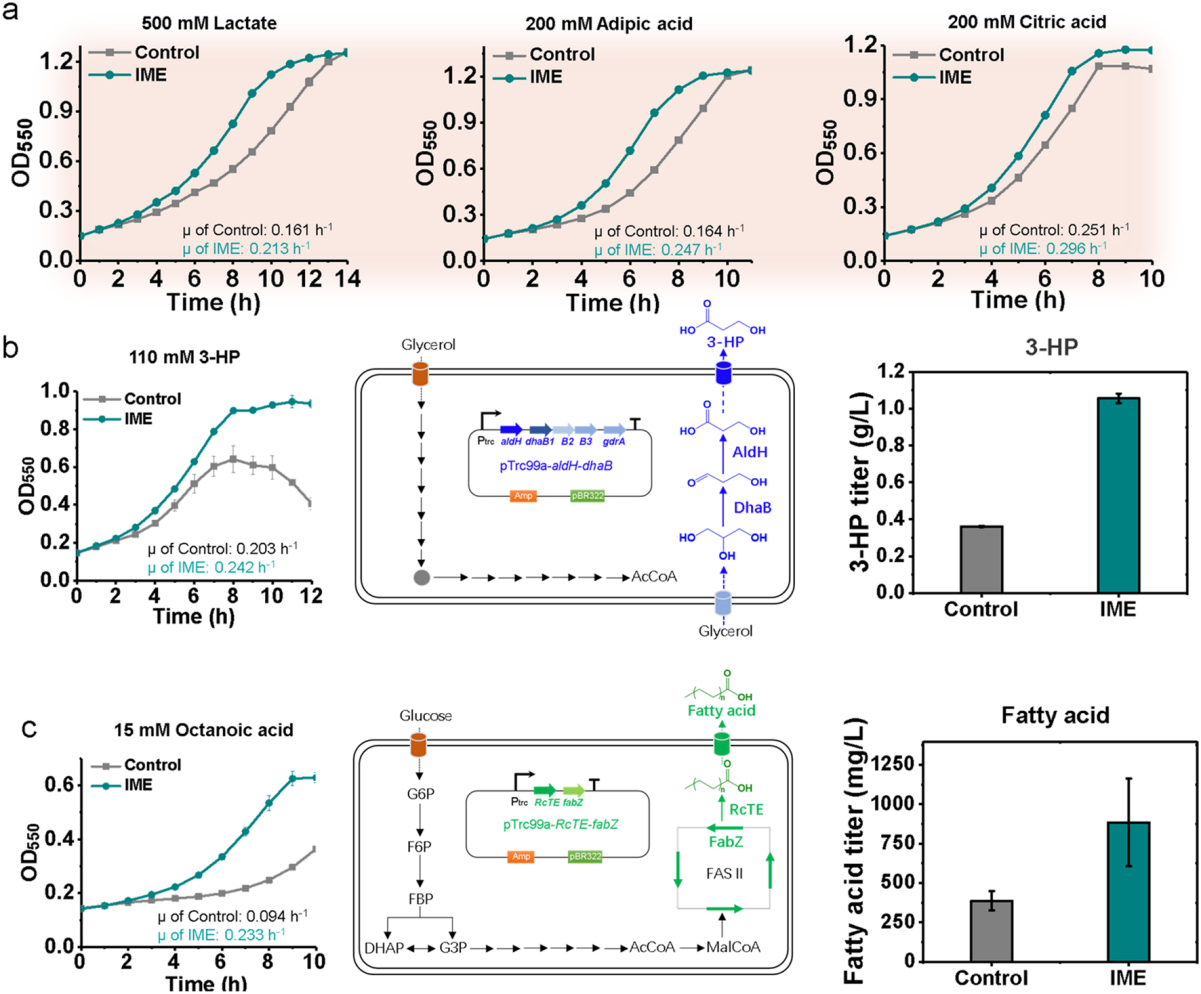
IME improved tolerance to inhibitory organic acids and production of organic acids. **a** IME had improved tolerance to organic acids. Growth curves were recorded in MOPS + 2% glucose medium with different inhibitors in a clear bottom 96-well plate at 37 °C with an initial pH 7.0. Error bars indicate standard deviation of at least three biological replicates. **b** IME had improved tolerance to 3-HP and increased 3-HP production. Both control and IME strains with pTrc99a-dhaB-aldH plasmid were cultivated in MOPS + 2% (w/v) glycerol medium in shake flasks at 37 °C, initial pH 7.0 for 48 h. **c** IME showed improved tolerance to octanoic acid and increased fatty acids production. Both strains with pXZ18Z plasmid harboring the genes encoding RcTE and *E. coli* 3-hydroxy-acyl-ACP dehydratase (FabZ) were cultivated in MOPS + 2% (w/v) glucose medium in shake flasks at 30 °C, initial pH 7.0 for 48 h. Error bars indicate standard deviation of at least three biological replicates. 3-HPA, 3-hydroxypropionaldehyde; 3-HP, 3-hydroxypropionate; DhaB, glycerol dehydratase; AldH, aldehyde dehydrogenase; RcTE, Ricinus communis thioesterase. FabZ, 3-hydroxy-acyl-ACP dehydratase

Lactic acid is a representative organic acid which contains both carboxylic acid group and hydroxyl group, and can serve as an important food additive in food industry, as well as precursor for manufacturing biodegradable and eco-friendly polylactic acid (PLA) polymers [54]. During challenge with 500 mM lactic acid (~ 45 g/L), μ of IME strain (0.213 h^−1^) increased by 33% over the control strain (0.161 h^−1^, *P* = 0.0075) (Fig. 5a). Adipic acid has been used as a food ingredient as a flavorant and gelling aid, as well as monomer for production of nylon by a polycondensation reaction with hexamethylene diamine [37]. In the presence of 200 mM adipic acid (~ 29.2 g/L), μ of IME strain (0.247 h^−1^) increased by more than 50% over the control strain (0.164 h^−1^, *P* = 0.008) (Fig. 5a). Citric acid is a tricarboxylic acid and used in flavor foods, beverages, insecticides and disinfectants. IME showed a roughly 15% increase (P = 0.003) in μ relative to the control strain when challenged with 200 mM citric acid (~ 38.4 g/L) (Fig. 5a). 3-Hydroxypropionate (3-HP) is an important platform chemical, ranked in the list of top 12 value added chemicals from biomass by US Department of Energy (DOE) [55]. During challenge with 110 mM 3-HP (~ 10 g/L), μ of IME strain (0.242 h^−1^) increased by 19% over the control strain (0.203 h^−1^, *P* = 0.001) (Fig. 5b). Moreover, fatty acids are attractive biorenewable chemicals and serve as precursors for synthesis of numerous molecules such as alkanes, methyl ketones and fatty alcohols. However, fatty acids are inhibitory to the biocatalyst [56]. Here, we found that IME effectively enhanced tolerance to fatty acids: in the presence of 15 mM (~ 2.2 g/L) octanoic acid, μ of IME strain (0.233 h^−1^) increased by ~ 150% over the control strain (0.094 h^−1^, *P* < 0.001) (Fig. 5c).

Next, we continued to evaluate whether IME improves synthesis of organic acids due to improved tolerance, and first applied it to 3-HP bioproduction. For construction of 3-HP biosynthetic pathway, the glycerol dehydratase gene *dhaB* from *Klebsiella pneumoniae* [57] and aldehyde dehydrogenase gene *aldH* from *Ralstonia eutropha* [57] were recruited and cloned into pTrc99a empty plasmid (Fig. 5b). The resulting plasmid pTrc99a-*dhaB-aldH* was subsequently transformed into both control and IME strains. When cultured in MOPS medium with 2% (wt/v) glycerol under shake-flasks, IME strain with pTrc99a-*dhaB-aldH* produced 1057 ± 26 mg/L of 3-HP in 48 h, which is ~ twofold higher than the titer from the control strain with the same plasmid (362 ± 3 mg/L) (*P* = 0.007) (Fig. 5b). Besides 3-HP, we also applied IME strain to bioproduction of fatty acids. Specifically, a plasmid pXZ18Z harboring *Ricinus communis* thioesterase was employed [58, 59]. When characterized in MOPS medium supplemented with 2% (wt/v) glucose, IME strain with pXZ18Z plasmid produced approximately 884 ± 278 mg/L of fatty acids in 48 h (Fig. 5c), which is 128% higher than the corresponding control strain (387 ± 61 mg/L) (*P* < 0.001).

### The IME strain can be efficiently inhibited by the commonly used antibiotics

**Fig. 6:**
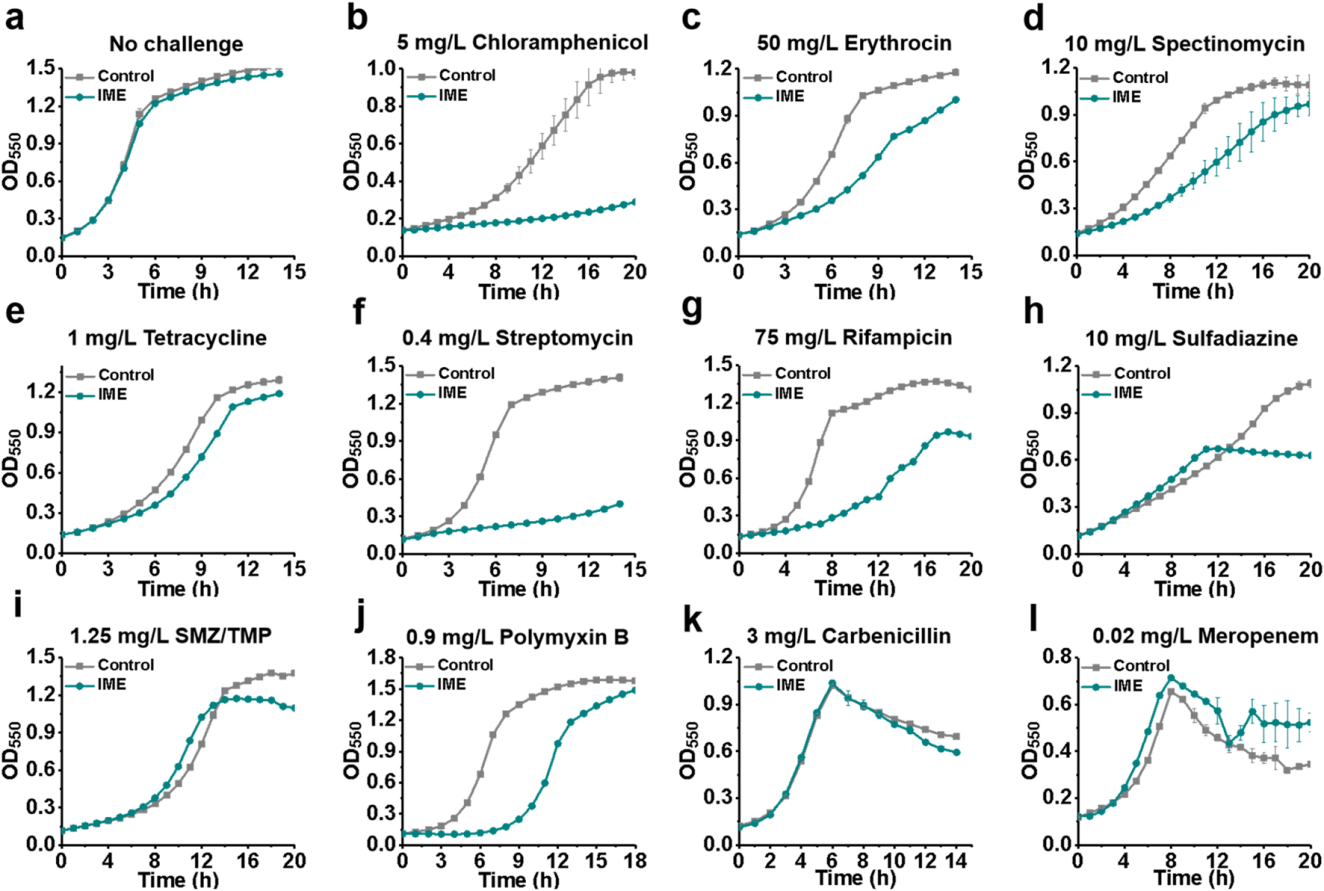
Sensitivity of the IME strain to various antibiotics. **a** IME and control strains showed no growth differences in the absence of antibiotics. The IME strain had increased sensitivity to **b** chloramphenicol; **c** erythromycin; **d** spectinomycin; **e** tetracycline; **f** streptomycin; **g** rifamycin; **h** sulfadiazine; **i** combination of sulfamethoxazole (SMZ) and trimethoprim (TMP); **j** polymyxin B; **k** carbenicillin; **l** meropenem. Growth curves were recorded in MOPS + 2% glucose medium with different antibiotics in a clear bottom 96-well plate at 37 °C, initial pH 7.0. Error bars indicate standard deviation of at least three biological replicates

Although the IME strain exhibits improved tolerance to adverse environmental conditions and industrially relevant chemicals, its uncontrolled use may pose the potential risk of developing undesirable drug resistance. Antibiotics are the most powerful weapons for killing bacteria, and can be classified into distinct types according to their inhibition mechanisms [60] (Additional file 1: Figure S5). The IME strain showed no growth difference as compared with the control strain in the absence of antibiotics (Fig. 6a).

We first tested the sensitivity of the IME strain to ribosome inhibitors, as protein synthesis represents one of the major inhibition targets for antibiotics. Chloramphenicol is a broad-spectrum antibiotic that binds to the 50S subunit of bacterial ribosomes and, thus, interferes with the bacterial protein synthesis [61]. In the presence of 5 mg/L chloramphenicol, the control strain showed the highest OD_550_ value of 1.0 in 16 h. However, the IME strain grew extremely slow and its OD_550_ value reached only 0.2 in 16 h (Fig. 6b). Erythromycin is another 50S ribosome inhibitor. When cultivated in the presence of 50 mg/L erythromycin, the μ of IME strain (0.156 h^−1^) decreased by 67% as compared to that of the control strain (0.261 h^−1^, *P* < 0.01) (Fig. 6c). Besides 50S ribosome inhibitors, we further evaluated the sensitivity of IME strain to 30S ribosome inhibitors. When exposed to spectinomycin (10 mg/L), tetracycline (1 mg/L), and streptomycin (0.4 mg/L), the IME strain showed a 62%, 30%, and 64% lower μ than the control strain, respectively (Fig. 6d-–f). These results demonstrate that the IME strain showed an obvious increase in its sensitivity to protein synthesis inhibitors.

We also assessed sensitivity of the IME strain to other types of antibiotics. Rifamycin is the most clinically important and extensively studied class of antibiotics that inhibits bacterial RNA polymerases [62]. After cultivation with 75 mg/L rifamycin, the μ of IME strain (0.090 h^−1^) decreased by 68% as compared to that of the control strain (0.280 h^−1^, *P* = 0.006) (Fig. 6g). Sulfonamides represent a class of antimicrobial drugs that inhibit bacterial folic acid synthesis [63]. We challenged the strains with 10 mg/L sulfadiazine, and found that the growth of IME strain terminated much earlier (11 h vs. 20 h) than that of the control strain; the maximum cell mass (OD_550_ ~ 0.7) achieved for the IME strain was 36% lower than that for the control strain (OD_550_ ~ 1.1) (Fig. 6h). A similar phenomenon was observed in the presence of sulfamethoxazole and trimethoprim combination (SMZ–TMP) (Fig. 6i). Polymyxins bind to lipopolysaccharide and disrupt the outer membrane of gram-negative bacteria [64]. Although the IME strain strengthened cell membrane, polymyxins still efficiently inhibited the growth of IME strain. In the presence of 0.9 mg/L of polymyxin B, the control strain exhibited a lag phase that lasted for 4 h. However, the lag phase doubled to 8 h for the IME strain (Fig. 6j). We speculated that this might be caused by that (*S*)-2,3-oxidosqualene mainly locates in the inner membrane of the IME strain. Beta-lactam antibiotics can destroy bacteria by interfering with cell wall synthesis [65]. We challenged the strains with 3 mg/L carbenicillin or 0.02 mg/L meropenem, and found that the IME strain had no tolerance (Fig. 6k, l). All these results demonstrate that IME strain can be effectively inhibited by the most commonly used antibiotics, showing no undesirable drug resistance.

## Discussion

Traditional tolerance engineering efforts of *E. coli* heavily rely on exploitation of the potential of itself alone [17–19], here we sought to introduce a heterologous lipid, (*S*)-2,3-oxidosqualene, into *E. coli*. For the first time, this non-native and eukaryotes-featured chemical was found to greatly alter the membrane properties, including decreasing the cell membrane leakage and increasing intracellular ATP content of *E. coli*, and finally improve *E. coli* robustness.

Here we introduced the heterologous SQS and SMO enzymes and further activated the native MEP pathway for enhancing (*S*)-2,3-oxidosqualene synthesis. As MEP pathway is often tightly regulated in *E. coli* [36] and (*S*)-2,3-oxidosqualene represents a eukaryotic molecule, replacement of the MEP pathway with MVA pathway in *E. coli* [66] can be performed to further increase the synthesis of (*S*)-2,3-oxidosqualene [67, 68]. In addition, adaptive laboratory evolution (ALE) can serve as another strategy to further improve biosynthesis of (*S*)-2,3-oxidosqualene through beneficial mutations [69], given that Reyes et al. had employed the ALE to improve carotenoids production in *S. cerevisiae* by exploiting the antioxidant properties of carotenoids [70].

In this study, the IME strain showed an increase in intracellular ATP content when challenged with stresses such as H_2_O_2_. We speculated that this phenomenon is associated with the decrease in cell membrane leakage due to integration of (*S*)-2,3-oxidosqualene. Specifically, in *E. coli*, the electrochemical concentration gradient of protons across inner membrane is employed to generate ATP through ATP synthase [71]. In the control strain, the presence of membrane-damaging chemical H_2_O_2_ would greatly disrupt this gradient of protons, thus compromising the ATP synthesis. In contrast, under the same condition, integration of (*S*)-2,3-oxidosqualene led to a significant decrease in cell membrane leakage, which contributes to maintaining the gradient of protons and ATP synthesis.

Prior studies have been performed before to improve the robustness of *E. coli* membranes by introducing non-native membrane compounds. In particular, Caforio et al. introduced the archaeal ether lipid biosynthesis genes in *E. coli*, constructed a stable hybrid heterochiral membrane and revealed that it exhibited a higher tolerance to l-butanol [72]. Santoscoy and Jarboe exploited the promiscuity of squalene hopene cyclase, and introduced it into *E. coli* to produce cholesterol-like molecules, which efficiently improves *E. coli* robustness and production capacity [73]. However, these strategies frequently take effect to only one certain stress [20–22, 74], our study showed that integration of the heterologous (*S*)-2,3-oxidosqualene enabled *E. coli* improved tolerance to multiple adverse stresses (Figs. 3–5), which is not easily accessible through current engineering methods. These adverse stresses include a variety of adverse environmental conditions of industrial relevance and can impact the process cost. Specifically, the increased thermotolerance could be useful in industrial applications by reducing cooling water usage and decreasing the probability of contamination. Increased tolerance to low pH can be applied to reducing base addition for maintaining the optimal pH. In addition, the increased osmotic pressures tolerance can be applied to the conditions with high-salt and high-sugar challenges, i.e., increased tolerance to high sugar can be applied to cultivation *E. coli* with a high initial sugar concentration for minimizing sugar feeding. In addition to adverse conditions, IME improved tolerances to inhibitors in feedstocks, including lignocellulose hydrolysate, suggesting its potential for using cheap and renewable feedstocks. In addition, IME increased tolerance and production of inhibitory bioproducts such as organic acids, which provides a better alternative chassis to naturally existing *E. coli* for metabolic engineers and synthetic biologists.

Here we also observed that the IME strain showed increased sensitivity to most of antibiotics. We speculated that this would be due to that integration of heterologous (*S*)-2,3-oxidosqualene into the cell membrane might affect the numbers and activities of antibiotics transporters [75]. In particular, up-regulation of antibiotics importers and/or down-regulation of antibiotics efflux pumps would contribute to the increased intracellular concentration of antibiotics in IME strain [76], and thus enabled IME strain to increase sensitivity to these antibiotics.

In summary, consistent with our initial plan to construct a super-robust *E. coli* chassis, like Iron Man, the IME not only has super strength and durability (increasing membrane integrity) to withstand damage (resisting to heat, high pressure, ROS, toxic feedstocks and inhibitory products), but also has super power (higher ATP energy) and abilities (production of a variety of inhibitory chemicals). More importantly, IME can be easily controlled by human beings.

## Methods

Detailed materials and methods can be found in the online supporting material.

### Strains and plasmids

All plasmids and strains used in this study are listed in Table S1. All strains are derivatives of *E. coli* MG1655. One-step recombination (FLP–FRT) [77] and CRISPR–Cas9 method [78] were used for chromosomal editing. Gene screening was performed by protein soluble expression in *E. coli*. Candidate genes were cloned into the P1 site of pCDF-duet1 vector, and introduced to *E. coli* strain BL21(DE3), under 0.1 mM IPTG induction to test their solubility. Gene of *tErg9*, *smo* was inserted, respectively, at *mgsA*, *pta* site and controlled by constitutive promoter M1–93 [79]. The native promoter of *idi* gene was replaced with M1–46 promoter.

### Growth conditions and characterization

All tolerance experiments were performed in 200μL MOPS + 2% (wt/v) glucose medium [80] in clear-bottom 96-well plate at 37 °C with an initial pH of 7.0. High temperature tolerance was assessed at 42 °C, all other tolerance experiments were performed at 37 °C. Specific growth rate μ (h^−1^) was calculated by fitting the equation OD_550,t_ = OD_550,0_ e^μt^ to the exponential growth phase. All estimated μ values had an R^2^ of at least 0.95.

### Protein expression and gel electrophoresis

The recombinant strains BL21(DE3)::tErg9, BL21(DE3)::SMO_*Mc*_ were grown in LB medium, Antibiotics were added with respect to the plasmid(s) present in the strain(s). The cultures were induced with 0.1 mM IPTG at ~ 0.6 OD_600_. The cells were harvested at 4 h after induction, and centrifuged at 10,000 g and 4 °C for 5 min. The cell pellets were washed twice with 100 mM potassium phosphate buffer (pH 7.0) and resuspended in the same buffer. The cells were disrupted using a French Pressure Cell (FA-078A, Thermo Electron Corp.; Waltham, MA) at1,250 psi. The cell lysates were centrifuged at 25,000 g for 30 min and the supernatants were used for SDS–PAGE. Coomassie Brilliant Blue R-250 was used to stain the proteins. The recombinant protein band intensities on SDS–PAGE were determined using Scion image analyzing software (Scion Corp., Frederick, MD).

### Extraction and detection of (*S*)-2,3-oxidosqualene

(*S*)-2,3-oxidosqualene were extracted by a modified Christoph Müller method [81]. Briefly, 10 mg of mid-log phase *E. coli* cells were collected, washed twice with 1 × phosphate-buffered saline, and mixed with 1 mL 2 M sodium hydroxide (NaOH). Flood the mixture with nitrogen and vortex vigorously for 1 min, this mixture was subsequently placed at 70 °C for 1 h for saponification. Then allow the suspension cool to room temperature and transferred to a fresh tube, 650 µL methyl-tert-butylether (MtBE) and 100 µL of internal standard (5α-cholestane in MtBE, 10 µg/mL) were added. Next, this mixture was vigorously hand vortexed and centrifuged, and the upper organic layer was transferred to a fresh tube. The lysed cell suspension was extracted once more with another 750 µL of MtBE. The initial and the second organic layers were combined and dried under nitrogen. Then, 850 µL of MtBE was added to redissolve the dried powder. Subsequently, 100 µL of cholesterol standard (10 µg/mL) and 50 µL of MSTFA/TSIM (9:1) (i.e. a combination of N-methyl-N-trimethylsilyltrifluoroacetamide (MSTFA) with 10% (vol/vol) N-trimethylsilylimidazole (TSIM)) were added to the sample, which was then placed at 22 °C for at least 0.5 h before being analyzed by GC–MS [82].

### Membrane Characterization

Membrane integrity was analyzed by SYTOX green (Invitrogen) staining [37], membrane fluidity was analyzed by 1,6-diphenyl-1,3,5-hexatriene (DPH) (Invitrogen) [38].

### Intracellular ATP content characterization

The intracellular ATP content was measured using an ATP assay kit (Beyotime) according to the manufacture’s introductions.

### Production of 3-HP or fatty acid using the IME strain

For 3-HP production, the overnight culture was inoculated into fresh M9 + 2.0% (wt/v) glycerol medium with an initial OD_550_ = 0.1. The inducer isopropyl-β-D-thiogalactopyranoside (IPTG) and the coenzyme vitamin B_12_ were added at a final concentration of 0.1 mM and 4 μM, respectively. Cultures were grown at 37 ℃ and 220 rpm for 72 h, and the concentration of 3-HP was determined by HPLC as described following. For fatty acid production, the overnight culture was inoculated into fresh MOPS + 2.0% (wt/v) glucose medium with an initial OD_550_ = 0.1. As the OD_550_ reached ~ 0.6, 0.1 mM IPTG was added. Cultures were grown at 30 ℃ and 220 rpm for 72 h. SCFA was extracted and analyzed using GC–MS [83]. LCFA was extracted and analyzed using GC–MS [58].

### Analytical methods of cells and for the fermentation products

The cell concentrations were measured in a 10-mm path-length cuvette using a double-beam spectrophotometer (Lambda 20, Perkin-Elmer; Norwalk, CT). One unit of absorbance at 600 nm corresponded to 0.333 g dried cell mass per liter. The concentration of 3-HP was determined by HPLC using refractive index (RI) detector (HPLC, Agilent 1100 series, USA). Supernatants, obtained by centrifugation of culture samples at 10,000 g for 5 min, were filtered through 0.2 mM Tuffryn-membranes (Acrodisc, Pall Life Sciences) and eluted through a 300 mm 7.8 mm Aminex HPX-87H (Bio-Rad, USA) column at 35 °C using 5 mM H_2_SO_4_. The flow rate and the flow cell temperature were set at 0.5 ml min^−1^ and 60 °C, respectively. The injection volume was 20 µl.

### Statistical analysis

The two-tailed *t* test method was employed to analyze the statistical significance of all the data in this study.

### Supplementary Information

**Additional file1: Figure S1.** SDS–PAGE analysis of the soluble expression of tErg9 (predicted Mw ~49 kDa) (left) and SMO (predicted Mw ~48 kDa) (right) in *E. coli* BL21 (DE3) host strain. **Figure S2.** SMO from *Methylococcus capsulatus* shares a 22% primary protein sequence identity with ERG1 from *Saccharomyces cerevisiae*. **Figure S3.** MS information related to detection of (*S*)-2,3-oxidosqualene using gas chromatography–mass spectrometry. **Figure S4.** Plasmid-overexpression of Idi of MEP pathway and heterologous SMO and SQS enzymes compromised the growth of *E. coli *strain. Growth curves were recorded in MOPS + 2% glucose medium without (left) and with 0.2 mM IPTG (right) in a clear bottom 96-well plate at 37 °C, pH 7.0. **Figure S5.** Inhibitory mechanisms of different types of antibiotics. **Table S1.** Strains and plasmids used in this study. **Table S2.** Primers and sequence used in this study.

## Data Availability

The corresponding author is willing to provide the data related to this manuscript upon reasonable request.
