# Supplementary material for: Integration of (S)-2,3-oxidosqualene enables E. coli to become Iron Man E. coli with improved overall tolerance
Source: Biotechnol Biofuels Bioprod. 2023 Dec 10;16:191. doi: 10.1186/s13068-023-02444-7 (PMC10710732; doi:10.1186/s13068-023-02444-7)
Supplement: Supplementary file 1 — Additional file1: Figure S1. SDS–PAGE analysis of the soluble expression of tErg9 (predicted Mw ~49 kDa) (left) and SMO (predicted Mw ~48 kDa) (right) in E. coli BL21 (DE3) host strain. Figure S2. SMO from Methylococcus capsulatus shares a 22% primary protein sequence identity with ERG1 from Saccharomyces cerevisiae. Figure S3. MS information related to detection of (S)-2,3-oxidosqualene using gas chromatography–mass spectrometry. Figure S4. Plasmid-overexpression of Idi of MEP pathway and heterologous SMO and SQS enzymes compromised the growth of E. coli strain. Growth curves were recorded in MOPS + 2% glucose medium without (left) and with 0.2 mM IPTG (right) in a clear bottom 96-well plate at 37 °C, pH 7.0. Figure S5. Inhibitory mechanisms of different types of antibiotics. Table S1. Strains and plasmids used in this study. Table S2. Primers and sequence used in this study. [file 13068_2023_2444_MOESM1_ESM.docx]

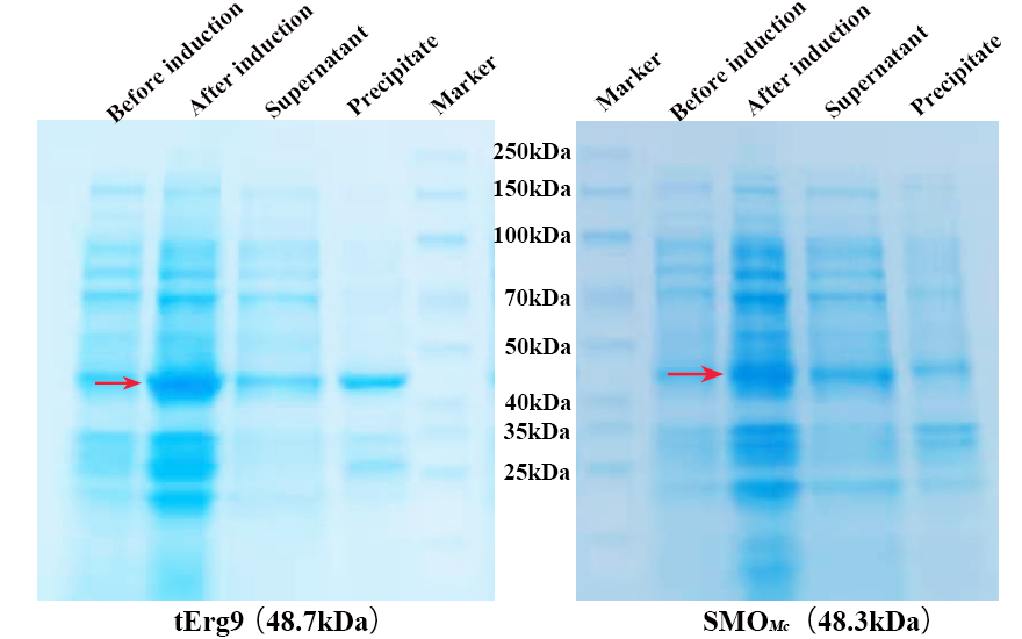


Fig. S1: SDS-PAGE analysis of the soluble expression of tErg9 (predicted Mw ~49 kDa) (left) and SMO (predicted Mw ~48 kDa) (right) in *E. coli* BL21 (DE3) host strain.


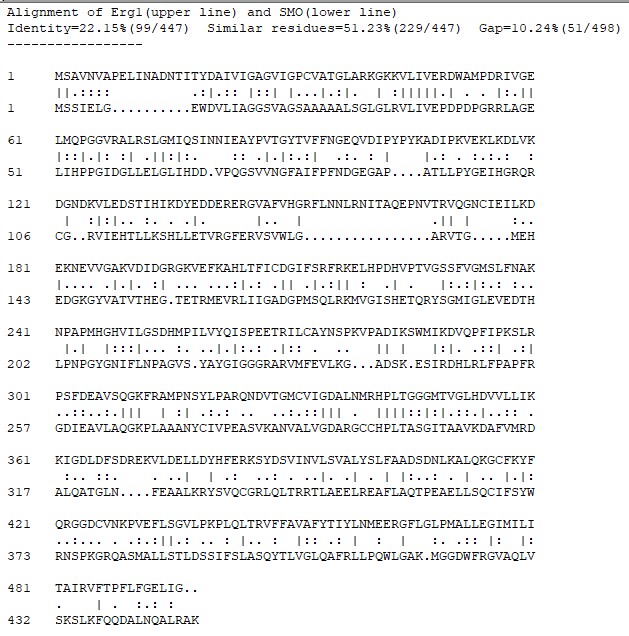


Fig. S2: SMO from *Methylococcus capsulatus* shares a 22% primary protein sequence identity with ERG1 from *Saccharomyces cerevisiae.*


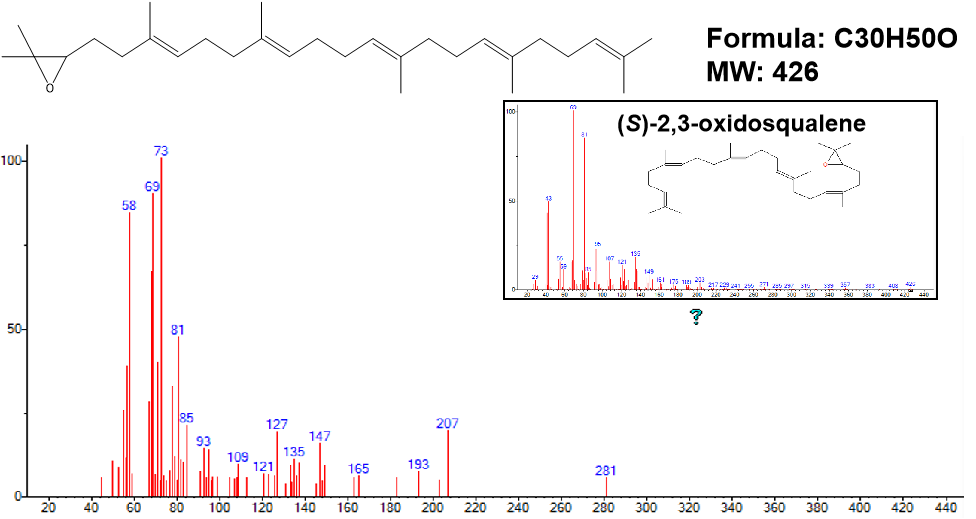


Fig. S3: MS information related to detection of (*S*)-2,3-oxidosqualene using gas chromatography-mass spectrometry.


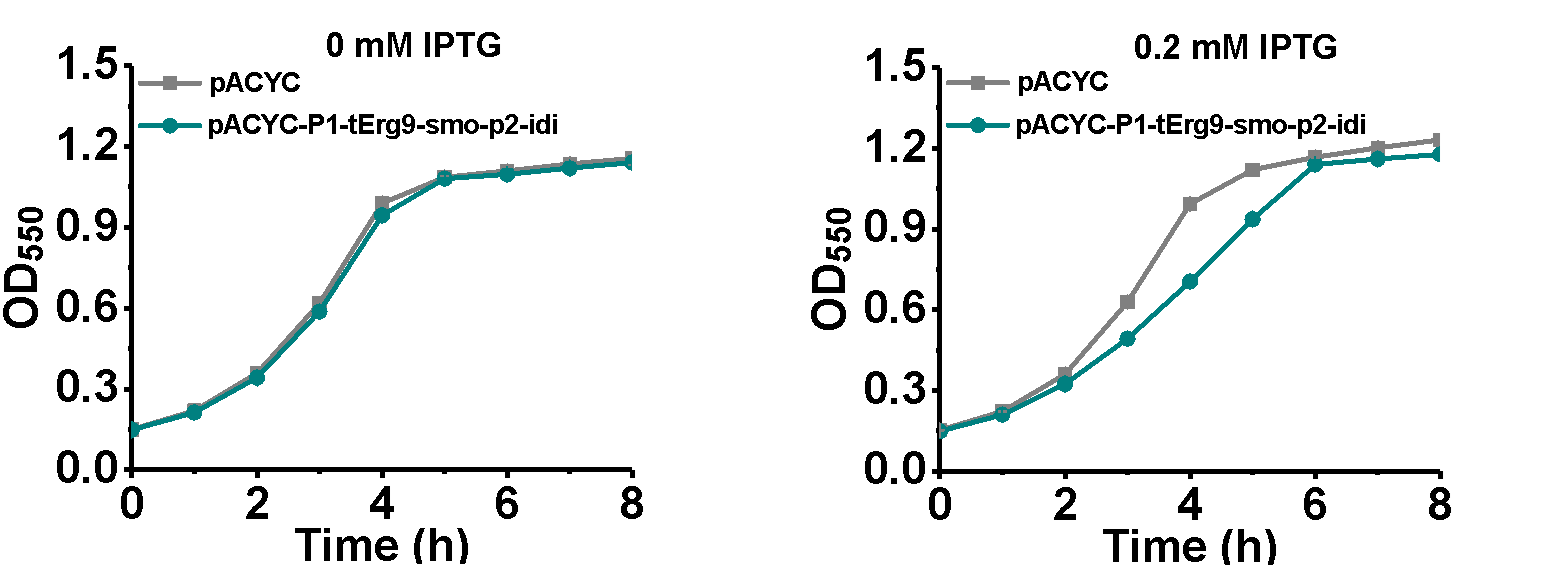


Fig. S4: Plasmid-overexpression of Idi of MEP pathway and heterologous SMO and SQS enzymes compromised the growth of *E. coli* strain. Growth curves were recorded in MOPS + 2% glucose medium without (left) and with 0.2 mM IPTG (right) in a clear bottom 96-well plate at 37 °C, pH 7.0.


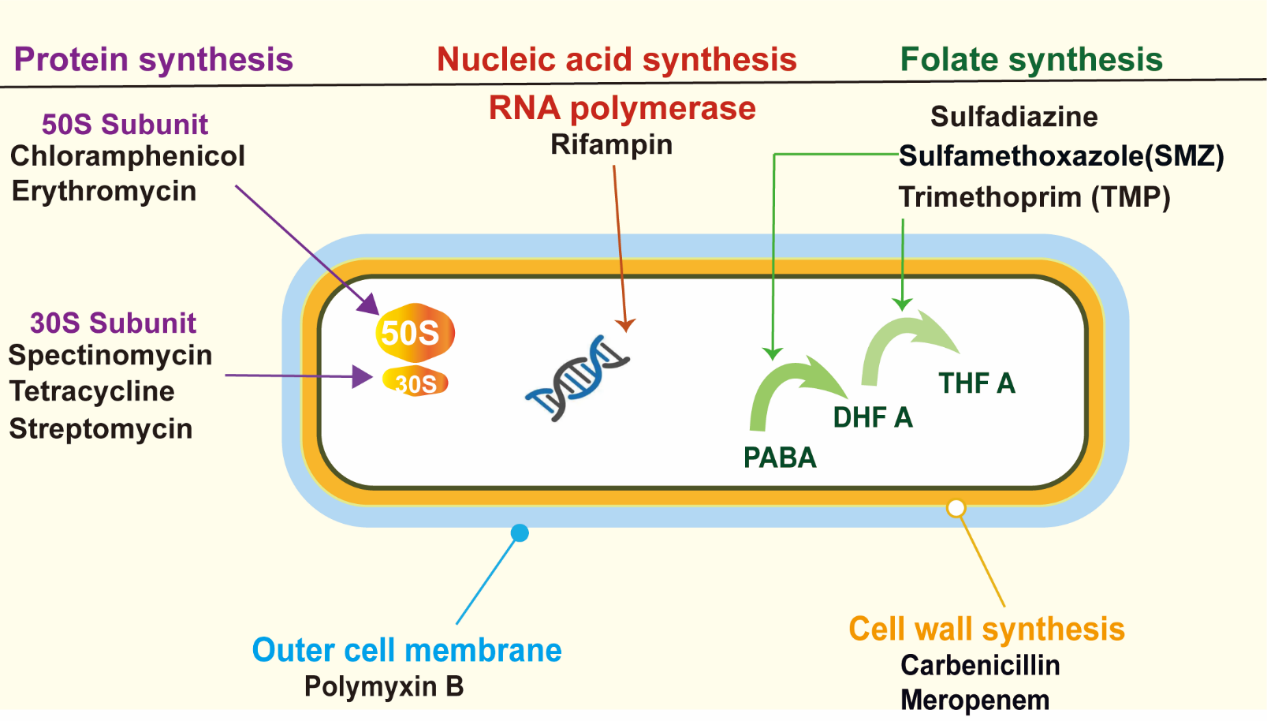


Fig. S5: Inhibitory mechanisms of different types of antibiotics

Table S1. Strains and plasmids used in this study

| Strains/plasmids | Genetic characterics | Source | | |
| --- | --- | --- | --- | --- |
| **Strains** |  |  | |  |
| MG1655 | Wild-type *E. coli* K1-12 strain | Lab collection | |  |
| Control | MG1655, Δ*mgsA,* Δ*pta*, M1-46-*idi* | This study | | |
| IME | MG1655, Δ*mgsA*::M1-93-*tErg9*, Δ*pta*::M1-93-*smo*, M1-46-*idi* | This study | | |
| **Plasmids** |  |  | | |
| pXZ18Z | pTrc99a-Ricinus communis thioesterase-fabZ, Amp^r^ | 23 | | |
| pTrc99a-*dhaB-aldH* | pTrc99a-glycerol dehydratase gene *dhaB* from *Klebsiella pneumoniae* and aldehyde dehydrogenase gene *aldH* from *Ralstonia eutropha* | 21 |  | |

**Table S2. Primers and sequence used in this study**

| Name | Sequence | Use |
| --- | --- | --- |
| MgsA-L-up | CAACACGCTGGCCGAAGT | Used for amplification of MgsA left homologous arm |
| MgsA-L-down | ATCCAGTCGCCGCATTTC |  |
| MgsA-L-KO-down | CTGATGAGCTGGGTGGAACGATCCAGTCGCCGCATTTCAA | Used with MgsA-L-up for amplification of MgsA left homologous arm for MgsA knock out |
| MgsA-R-up | CGTTCCACCCAGCTCATC | Used for amplification of MgsA right homologous arm |
| MgsA-R-down | CTCGCCATTACCTCAACG |  |
| M1-93-tErg9-up | TTGAAATGCGGCGACTGGATTTATCTCTGGCGGTGTTG | Used for amplification of M1-93 promoter that fused to tErg9 |
| M1-93-tErg9-down | AATTGTAATAGCTTTCCCATAGCTGTTTCCTGGTTTAAAC |  |
| tErg9-up | ATGGGAAAGCTATTACAATTGGC | Used for amplification fragment |
| tErg9-Cas9-down | CTGATGAGCTGGGTGGAACGTCAGTACTCTTCTTCTTGTTGGG | of tErg9 that fused to mgsA-R |
| MgsA-L-up1 | TCACATGAGGCCTGCCAG |  |
| tErg9-YZ-down | GCAGCTCTCTGATCACAGC | Used with mgsA-L-up1 for verification of tErg9 insertion |
| pta-L-up | TGACCAAAGAGTCTGGCCT | Used for amplification of pta |
| pta-L-down | GGTTTATCCTCTTTCGTTACCG | left homologous arm |
| pta-L-KO-down | AGCTGCGGATGATGACGAGAGGTTTATCCTCTTTCGTTACCG | Used with pta-L-up for amplification of pta left homologous arm for pta knock out |
| pta-R-up | TCTCGTCATCATCCGCAG | Used for amplification of pta |
| pta-R-down | GTCGTGAACAGCTGTACGC | right homologous arm |
| M1-93-SMO-up | GTAACGAAAGAGGATAAACCTTATCTCTGGCGGTGTTG | Used for amplification of M1-93 |
| M1-93-SMO-down | CCCAGTTCGATGCTGCTCATAGCTGTTTCCTGGTTTAAAC | promoter that fused to tErg9 |
| SMO-up | ATGAGCAGCATCGAACTGGG | Used for amplification fragment |
| SMO-Cas9-down | CTGCGGATGATGACGAGATTATTTAGCACGCAGCGCC | of tErg9 that fused to pta-R |
| pta-L-up1 | TGAGCGTTGACGCAATCA |  |
| SMO-YZ-down | GTCCGGTTCAACGATCAGAA | Used with pta-L-up1 for verification of SMO insertion |
| pTargetF-up | ACTAGTATTATACCTAGGACTGAG |  |
| pTargetF-mgsA-down | GTCCTAGGTATAATACTAGTAACGTCAACGCGATGTTGAGGTTTTAGAGCTAGAAATAGC | Used with pTargetF-up to amplify pTargetF-mgsA |
| pTargetF-pta-down | GTCCTAGGTATAATACTAGTGCTGATTCCGCTGCGGCCTTGTTTTAGAGCTAGAAATAGC | Used with pTargetF-up to amplify pTargetF-pta |
| IdI-M1-46-up | TCACTTGGTTAATCATTTCACTCTTCAATTATCTATAATGATGAGTGATCAGGAACACTTAACGGCTGAC |  |
| IdI-M1-46-down | CCCGTGGGAACTCCCTGTGCATTCAATAAAATGACGTGTTCCGTTTGCATAGCTGTTTCCTGGTTTAAAC | Used for the amplification fragment of Idi-M1-46-FRT-kana-FRT in the one-step recombination |
